# Case Report: Rare percutaneous coronary intervention for “right” main bifurcation

**DOI:** 10.3389/fcvm.2023.1287907

**Published:** 2023-11-23

**Authors:** Po-Hsueh Su, Cheng-Yu Ko, Cheng-Han Lee

**Affiliations:** ^1^ Division of Cardiology, Department of Internal Medicine, National Cheng Kung University Hospital, College of Medicine, National Cheng-Kung University, Tainan, Taiwan; ^2^ Division of Cardiology, Department of Internal Medicine, Madou Sin-Lau Hospital, Tainan, Taiwan

**Keywords:** congenital coronary anomalies, coronaries of anomalous origin, percutaneous coronary intervention, “right” main bifurcation, IVUS guided PCI

## Abstract

We presented the case of a patient with non-ST-elevation myocardial infarction with coronary arteries of an anomalous origin, an interarterial course of the LMCA, a unique wide-angle “right” main bifurcation lesion, and a high SYNTAX score. Management with contemporary PCI and imaging may be an alternative to surgery.

## Introduction

This was a rare case of patient with anomalous aortic origin of a coronary artery (AAOCA), an interarterial course of the LMCA, and a unique wide-angle “right” main bifurcation lesion (bifurcation of the RCA and LMCA) who presented with acute myocardial infarction. The patient was successfully managed using double guide catheters under the guidance of CCTA and IVUS.

## Case description

**Figure 1:**
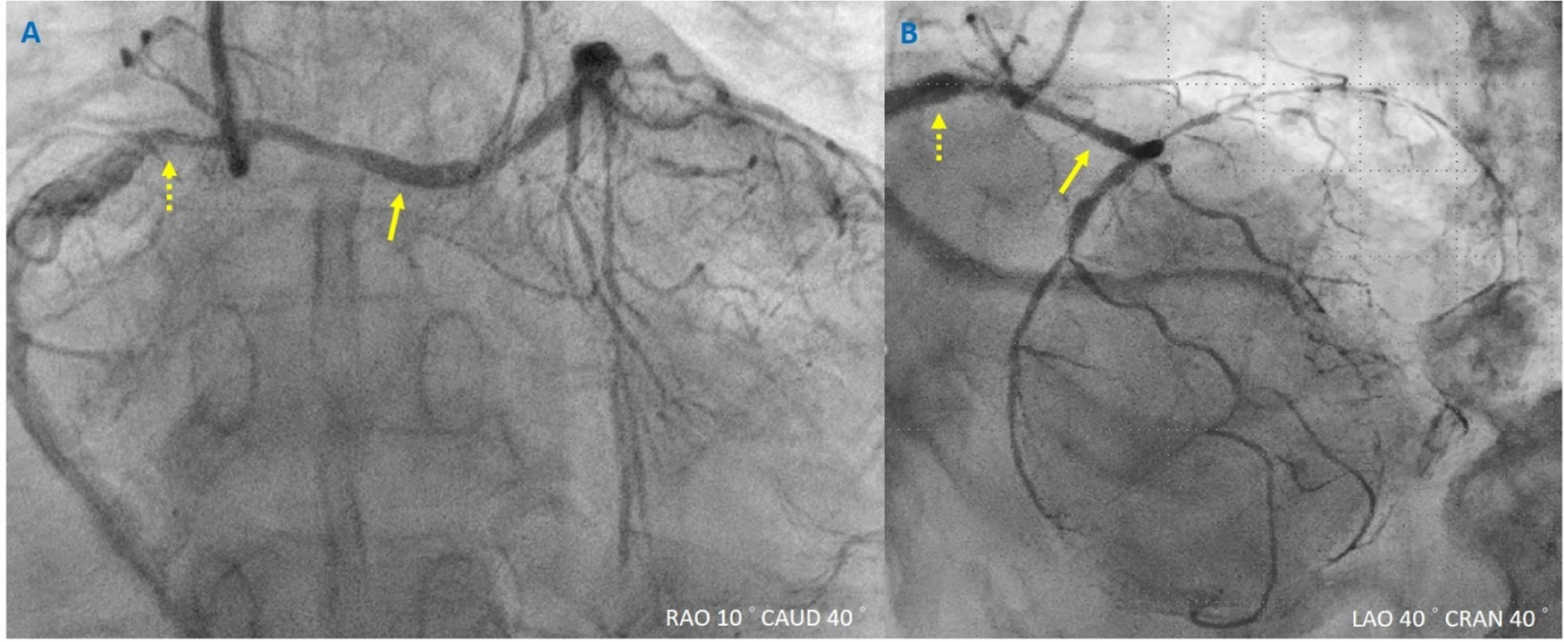
Coronary angiography for non-ST-elevation myocardial infarction. (**A,B**) Yellowish dashed arrow indicates the right coronary artery, and yellowish arrow indicates the left main coronary artery. Different views of anomalous aortic origin of a coronary artery with a common ostium.

**Figure 2:**
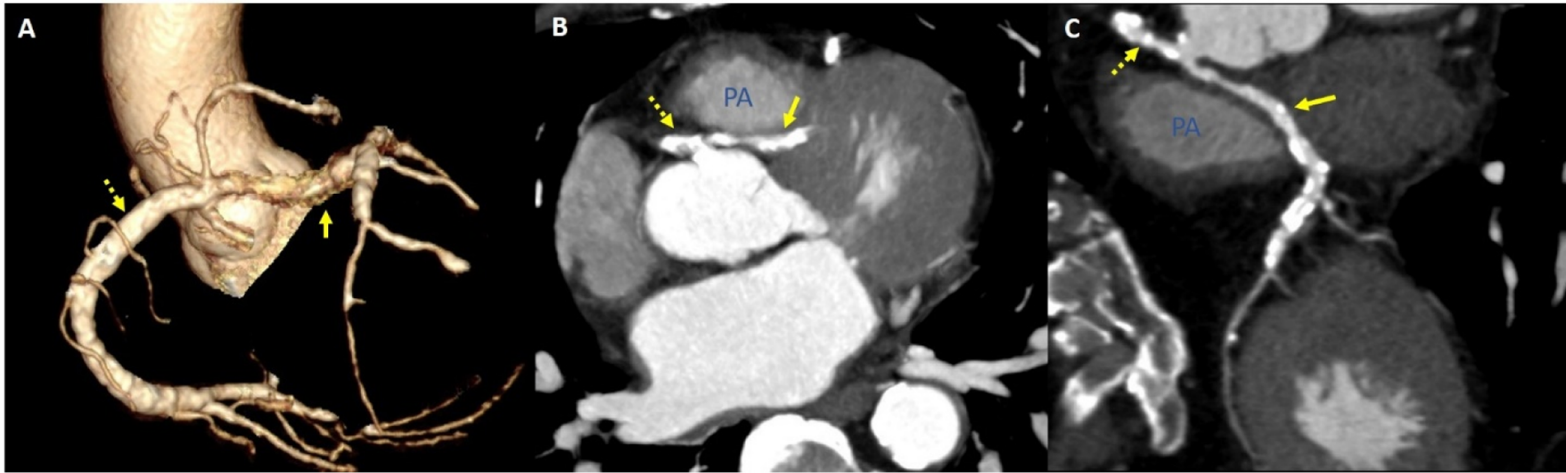
Coronary computed tomography angiography assessment for anomalous aortic origin of a coronary artery. (**A**) Reconstructive image revealed “right” main bifurcation. (**B,C**) Coronary computed tomography angiography showed a common ostium for the left main coronary artery and the right coronary artery, characterized by an interarterial course.

An 83-year-old man with diabetes mellitus and hypertension, without the history of coronary artery disease before, presented with chest tightness for several hours and received a diagnosis of non-ST-elevation myocardial infarction on the basis of troponin elevation and electrocardiography indicating ST depression in the lateral leads. Invasive coronary angiography showed a common origin of the right and left coronary arteries at the right coronary cusp with diffuse atherosclerosis and multiple stenotic lesions (Figure 1; Supplementary Video S1). For detailed anatomy, we performed coronary computed tomography angiography (CCTA), which revealed an anomalous origin of the left main coronary artery (LMCA) from the right sinus of Valsalva and an interarterial course between the aorta and the pulmonary artery; the “right” main bifurcation angle was close to 180° (Figure 2). This patient was diagnosed coronary artery disease at first time.

## Diagnostic assessment

On the basis of the anomalous origin of the LMCA with an interarterial course and a high SYNTAX score of 47, surgical intervention was recommended consistently with current guidelines (1). However, the patient refused surgery and chose percutaneous coronary intervention (PCI) because of his older age and frailty.

**Figure 3:**
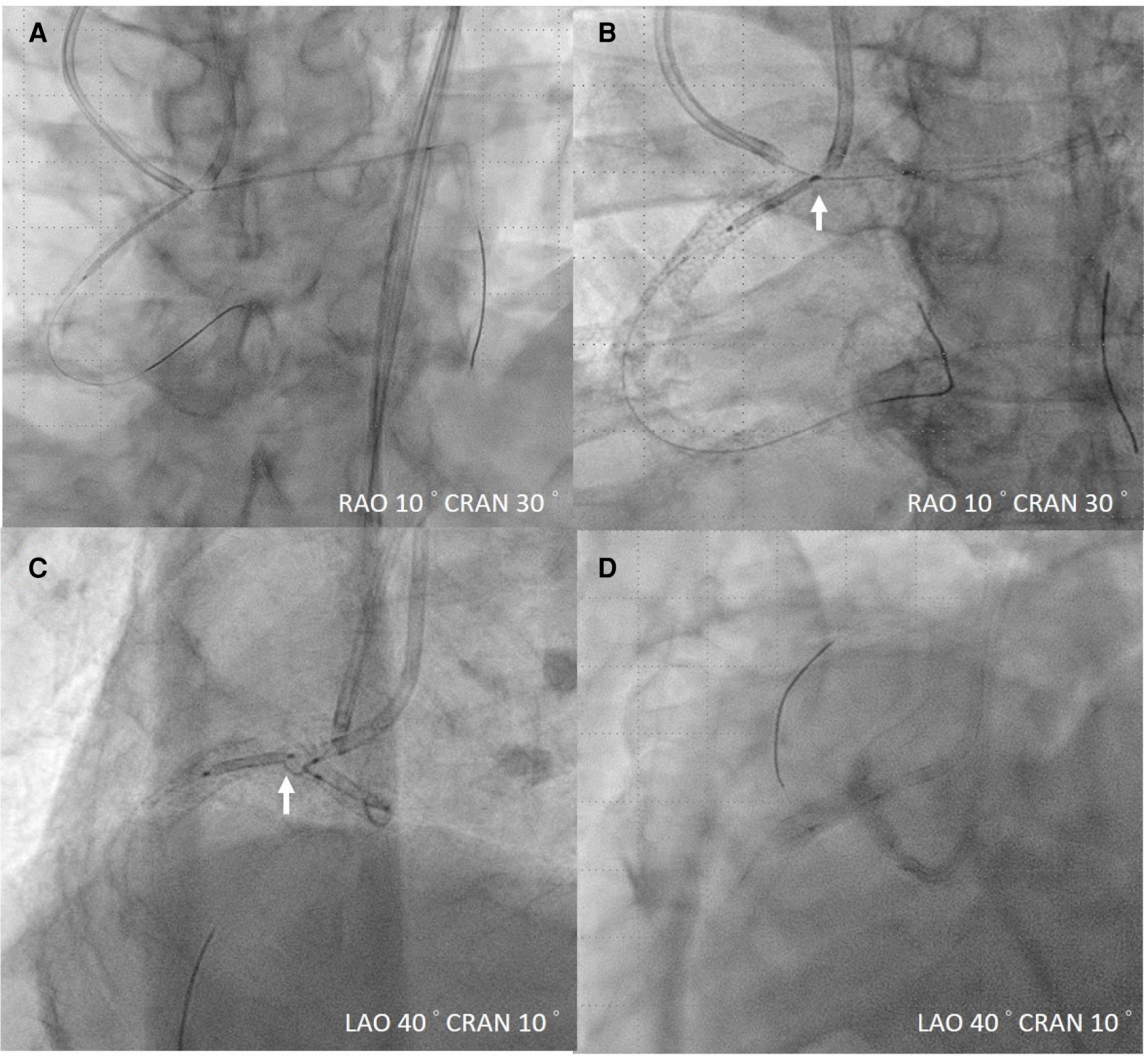
Management of AAOCA stenosis. (**A**) Intravascular ultrasound (IVUS)-guided stent deployment. (**B,C**) White arrow indicates the proximal mark of the right coronary artery stent, positioned close to the center of the proximal left main coronary artery stent, in accordance with IVUS guidance. (**D**) Kissing balloon technique for ostium bifurcation. AAPCA, anomalous aortic origin of a coronary artery.

**Figure 4:**
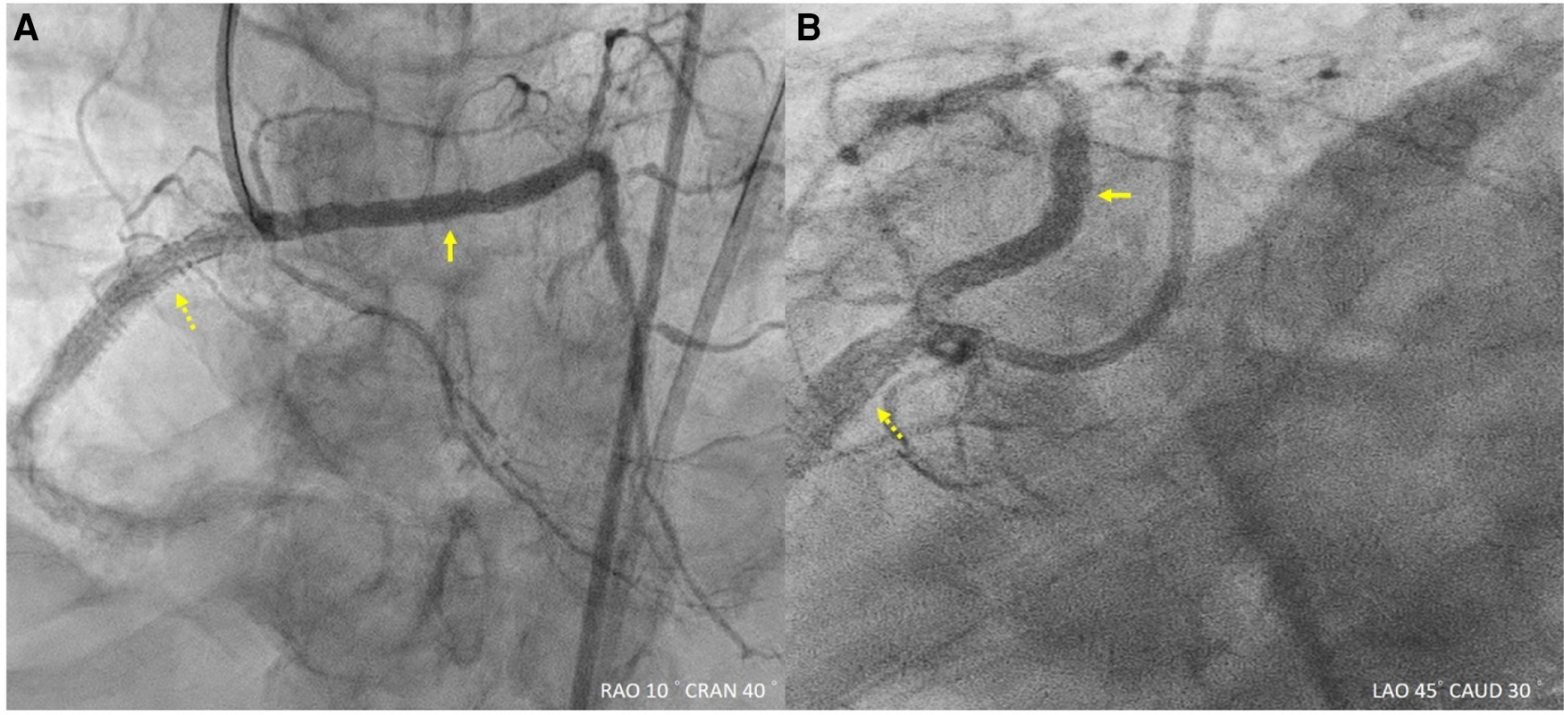
Final angiography. (**A,B**) Final angiography revealed improved flow.

Our strategy was to perform stenting with little protrusion of the stents into the common ostium. We would not like excessive stent protrusion because the common ostium was large, more than 6 mm, without significant stenosis. Due to the wide angle of the “right” main bifurcation and for more precise stent deployment, two guide catheters were used to engage the common ostium: a JR4 guide catheter and a SAL1 guide catheter (6 Fr; Medtronic, Minneapolis, MN, USA; Supplementary Video S2). After the insertion of coronary guidewires (Runthrough NS Hypercoat, Terumo, Tokyo, Japan; Fielder FC, Asahi Intecc, Seto, Japan) into the left anterior descending artery and the right coronary artery (RCA), we noted diffuse stenosis and calcification on intravascular ultrasound (IVUS) (Supplementary Video S3A and Video S3B). Under the guidance of IVUS and CCTA, a SYNERGY stent (3.5 mm × 38 mm; Boston Scientific, Marlborough, MA, USA) was deployed within the LMCA from LMCA orifice to distal part, before the bifurcation of LAD and LCX to cover diffuse LMCA lesions and the inter-arterial course. Two SYNERGY stents (4.5 mm × 24 mm and 5.0 mm × 12 mm) were deployed within the RCA from the orifice to middle part. (Figure 3; Supplementary Video S4). The LMCA was stented to cover the inter-arterial course of an anomalous coronary artery. The stents were used in both orifices with little protrusion to the common ostium. Afterward, the kissing balloon technique was used to ensure complete stent apposition in both orifices; to accomplish this, a noncompliance balloon catheter (4.0 mm × 15 mm; Conqueror, APT Medical, Shenzhen, China) was used for the LMCA and a noncompliance balloon catheter (5.0 mm × 12 mm; Emerge, Boston Scientific, Marlborough, MA, USA) was used for the RCA (Figure 3). The IVUS showed that the stents were well apposed without external compression on images, and the final angiography revealed improved flow (Figure 4; Supplementary Video S5). The interarterial course of an anomalous coronary artery was stented according to correspond coronary CTA. The patient was discharged several days after the procedure, without any recurrent symptoms. The patient was discharged smoothly several days after the procedure, and had no recurrent angina. The follow-up echocardiography one year later revealed preserved left ventricular ejection fraction and the patient still could walk with canes.

## Discussion

Although AAOCA affects at least 1 in 1,000 individuals, it is difficult to extrapolate the accurate estimate of its burden due to heterogeneous observational studies. One review article suggested that interarterial anomalous left coronary artery is even rare with a weighted prevalence of 0.03% from the observational data (2, 3). Most patients with AAOCA are asymptomatic (4). Natural history studies focusing on patients with untreated AAOCA are scarce (5). The optimal management of this condition remains controversial, particularly in patients with an interarterial course of an anomalous coronary artery, which is thought to increase the risk of serious complications such as angina pectoris, myocardial infarction, syncope, and ventricular tachycardia. A previously proposed pathophysiological mechanism suggests that the LMCA located between the aortic root and the pulmonary trunk can worsen the preexisting angulation of the coronary artery and reduce the diameter of the lumen in the proximal portion of the coronary artery during exercise (6). Surgery is recommended for AAOCA in patients with typical angina symptoms presenting with the symptoms of stress-induced myocardial ischemia in a relevant high-risk anatomical area (5).

Very few cases of patients with AAOCA and concomitant acute myocardial infarction have been reported, and no case report indicated an interarterial course with complex bifurcation lesions. Surgical intervention was recommended for our patient on the basis of current guidelines. One case was reported involving similar coronary anatomy with a common ostium arising from the right sinus of Valsalva (7); emergent PCI was performed for the RCA, but the procedure failed because of the complex anatomy involved.

Our patient had a high SYNTAX score but chose to receive PCI because of his older age and frailty. Surgical intervention was recommended on the basis of the current guidelines. Nonetheless, PCI using contemporary techniques, such as double guide catheters, and imaging techniques, such as IVUS and CCTA, may be alternatives to surgery.

## Conclusion

We presented the case of a patient with non-ST-elevation myocardial infarction with coronary arteries of an anomalous origin, an interarterial course of the LMCA, a unique wide-angle “right” main bifurcation lesion, and a high SYNTAX score. Management with contemporary PCI and imaging may be an alternative to surgery.

## Data Availability

The raw data supporting the conclusions of this article will be made available by the authors, without undue reservation.

## Supplementary material

SUPPLEMENTARY VIDEO S1

Diagnostic coronary angiography. During the diagnostic angiography performed for the right coronary artery by using a JR4 catheter, we discovered that the left main coronary artery originates from the right coronary cusp, sharing an ostium.

SUPPLEMENTARY VIDEO S2

Percutaneous coronary intervention for coronary artery stenosis. We used a JR4 guide catheter with a Fielder FC guidewire for the right common artery and a SAL1 guide catheter with a Runthrough NS Hypercoat guidewire for the left anterior descending artery.

SUPPLEMENTARY VIDEO S3

(**A**) Pre-IVUS image for RCA; (**B**) Pre-IVUS image for LMCA. After placing coronary guidewire to RCA and LMCA, diffuse stenosis and calcification were noted on intravascular ultrasound.

SUPPLEMENTARY VIDEO S4

Intravascular ultrasound–guided deployment of second RCA stent. The second RCA stent should be deployed such that the proximal mark of the RCA stent is near the center of the proximal left main coronary artery stent, as per intravascular ultrasound guidance. RCA, right coronary artery.

SUPPLEMENTARY VIDEO S5

Final coronary angiography. (**A,B**) Right and left anterior oblique angiographic views indicating favorable results.
